# Advanced Supervision of Smart Buildings Using a Novel Open-Source Control Platform

**DOI:** 10.3390/s21010160

**Published:** 2020-12-29

**Authors:** Peter Minarčík, Hynek Procházka, Martin Gulan

**Affiliations:** 1 Institute of Automation, Measurement and Applied Informatics, Faculty of Mechanical Engineering, Slovak University of Technology in Bratislava, 812 31 Bratislava, Slovakia; martin.gulan@stuba.sk; 2 Prosystemy, s.r.o. (Ltd.), 900 86 Budmerice, Slovakia; hynek.prochazka@prosystemy.sk

**Keywords:** smart buildings, supervision, global monitoring, open-source control platform, signal analysis

## Abstract

Gathering data and monitoring performance are at the heart of energy efficiency and comfort securing strategies in smart buildings. Therefore, it is crucial to present the obtained data to the user or administrator of such a building in an appropriate form. Moreover, evaluating the data in real time not only helps to maintain comfort, but also allows for a timely response from the user or operator to a possible fault. Continuous online monitoring and analysis of process behaviour, which is referred to as advanced supervision, is addressed in this paper by developing a procedure that will form an artificial operator autonomously supervising process. After introducing several techniques that are used for signal analysis, we propose an approach to advanced supervision of processes in smart buildings or other industrial control systems. The developed procedure is implemented on a control system platform that is particularly suitable for this purpose. Moreover, this platform includes a framework that provides support for the implementation of advanced control techniques and it is based on open-source tools, which is rarely seen in industrial applications. The developed advanced supervision procedure has been tested in simulation as well as in a practical case study using a real two-storey family house.

## 1. Introduction

Over the past decade, we have experienced an intense development of new smart devices that can connect to the internet and can be controlled remotely using applications. The network of such devices and other items embedded with sensors, electronics, software, and connectivity is referred to as the Internet of Things (IoT). IoT, smart home, energy consumption, remote control, global monitoring and supervision, or predictive maintenance are definitely some of the most discussed topics in the field of process control, whether in the residential, commercial, or industrial sector. In particular, the IoT is becoming a part of the daily operation of many industries. The support of smart buildings and homes is a related and cost-effective user-level IoT application [1,2]. Smart homes have become central in recent technology and policy discussions regarding energy efficiency, climate change, and the sustainability of buildings [3,4].

IoT-enabled smart building or home technologies refer to devices that provide some degree of digitally connected, automated, or enhanced services to building occupants [5]. They depend, to a great extent, on the use of an efficient and reliable control system. In addition to the academic research community, there is a large number of multinational, as well as smaller, industrial companies that are engaged in the development of control techniques and their implementation in practice. For instance, according to [6], some of the largest companies dealing with smart buildings and their management are Cisco, Honeywell, Johnson Controls, Schneider Electric, Emerson Electric, and Siemens [7]. Control systems that are offered by these vendors are not easily accessible to the general public though. On the contrary, the more affordable control platforms, such as Amit, Domat, Proview, Raspberry Pi, IndustrialShield, Unipi, etc., offer a good alternative. Nevertheless, these low-cost controllers miss or poorly support modern, advanced control techniques and design tools that are used in control engineering. This gap creates a challenge for developing new, ideally open-source and low-cost hardware and software tools that would provide advanced functionality for the monitoring and control of smart buildings.

### 1.1. Motivation

Smart buildings are not only buildings that can perform different types of operations for thermal comfort and occupants’ overall well-being based on various input data, but they are also able to provide information regarding their health state, level of stress, and wear of particular technological components. In addition, they can determine whether the operation is truly optimal and inform the end user about relevant statistical information. According to Mofidi and Akbar [8], in the past, the term intelligent or smart building merely referred to the implementation of automated operation and smart devices into buildings, without considering the needs of the occupant and the level of the interaction between the occupant and building itself. Nowadays, the occupants’ requirements and operation of smart buildings are well balanced. In order to maintain this balance, an appropriate and adequately complex system, which will provide data collection, global monitoring, and control of the given processes, has to be designed. The design of techniques and tools for intelligent monitoring and supervision of buildings has been subject of several recent works. For example, in [9], the authors present a building automation and control tool for remote and real-time monitoring of energy consumption in the building sector. The authors in [10] developed an IoT-based automated alert system for thermal comfort monitoring in buildings and, similarly, Wang et al. [11] proposed an integrated system for building structural health monitoring and early warning based on IoT. The role of intelligent supervision concept integrated into building predictive control has been comprehensively reviewed in [12].

Although there are numerous corporate Building Monitoring System (BMS) and/or Building Automation System (BAS) solutions available, advanced control techniques and algorithms have not been commonly applied in practice yet, neither have been advanced modelling environments, such as MATLAB by Mathworks or Scilab by INRIA used for development and practical deployment of these techniques. Moreover, an open-source, low-cost, and reliable control system platform for this purpose is practically missing. Therefore, the approach presented in this paper is realized on the basis of a novel Dynamic Control Unit (DCU) control system platform, which overcomes deficiencies of other systems in this way.

Despite numerous functionalities and advanced features, the DCU control system platform has not until now provided the advanced supervision of processes, whether these are thermodynamic processes in buildings or mechanical processes in systems in general. The motivation also stems from the industrial practice where experts and operators often need to manually monitor individual parts of each building in order to be able to observe any possible anomalies or unusual signal behaviour. Our objective is to design and implement the ability to automate this supervision process and, hence, to develop an adequate advanced supervision component as a part of the so-called Global Monitoring and Control System (GMCS). The GMCS will not only analyse the process and increase reliability of the system, but also improve knowledge about the controlled system. Within a larger set of functions designated as Advanced Supervision Server (ASS), the emphasis will be put on the analysis of a general signal (air/water temperature, pressure, etc.), which enables to detect and, consequently, determine the nature of a potential problem.

### 1.2. Signal Analysis and Anomaly Detection Methods

As already outlined, the focus of this paper is on the ASS part of the control platform and on the associated input-output signal analysis. These functions are used for the detection of anomalies occurring during a given process, including fault and illogical data trend detection.

This kind of problem is of interest to many research groups, and several techniques and methods have been developed for signal analysis and anomaly or fault detection. Not only time domain, but also frequency domain, are tool for signal analysis, and they are both suitable for solving these problems, as Majewski et al. [13] state. There are several methods that are utilised to obtain better insight into the basic nature of signal and its dynamics. For example, Elbi and Kizilkaya [14] focused on Fast Fourier Transform (FFT) and Short Time Fourier Transform (STFT), Wavelet Transform (WT), Gabor and Stockell transform, etc. It is widely known that the essential idea of the Fourier analysis is that every harmonic signal can be decomposed into an infinite sequence of sinusoids of different frequencies and, after the transformation into frequency domain, FFT creates a spectrum. STFT is a modification of FFT, which is able to window the analysed signal, thus removing the main disadvantage of FFT—not knowing the time when the specific frequency appears in the signal. However, there still remains a problem with the constant window width for analysis. On the contrary, WT has an advantage in its ability to change the width of the window dynamically. WT and its modifications are described in several works, e.g., Ren and Sun [15] advocate WT as an efficient tool for local signal analysis and, together with Shanoon entropy, were both used for structural damage detection. Li [16], on the other hand, combined wavelet analysis with Kalman filter for sudden fault detection. Sharma and Pachori [17], in turn, proposed an iterative decomposition of the Hankel matrix, enabling the decomposition of the mono-competent signal and following Hilbert transform, the amplitude is determined as well as frequency functions of each decomposed component. Another use of wavelet analysis can be found, for example, in [18,19,20]. Yeap et al. [21] and Sejdić et al. [22] summarise some of the advantages and disadvantages of the FFT and WT methods. It is demonstrated that FFT is fast, simple for implementation, accurate, and suitable for embedded systems, while its constant window width is the main disadvantage. On the contrary, wavelet analysis can serve as another example of variable resolution and it is accurate and immune to noise. However, it has a few disadvantages, such as high computational demands and filtering, leading to time delay.

There is a number of methods for anomaly detection that are based on different approaches. Mirnaghi and Haghighat [23] elaborated on a comprehensive review of their use in buildings and their Heating, Ventilation, and Air-Conditioning (HVAC) systems, who formulated three main approaches for fault detection and diagnosis, in particular, model-based methods, data-driven methods, and knowledge-based methods, which can also be combined to achieve a higher accuracy. Lughofer et al. [24] introduced another novel anomaly detection method, who proposed an approach that was based on a casual relation network for online anomaly detection in industrial environments. The outlier detection can significantly contribute to signal analysis and fault reveal. This problem was a point of interest, for example, for Smiti [25], who divided methods of this analysis into four groups—statistical detection methods, distance-based detection methods, density-based, and cluster-based detection methods. A comprehensive overview of research on anomaly detection can be found, e.g., in [26] or in [27]. Our approach of signal analysis and anomaly detection is based on the FFT/STFT technique, signal’s peaks detection, and the algorithm of cumulative sum.

## 2. A Novel Dynamic Control System Platform

The term control system platform represents the overall hardware and software setup for a standard building, as well as for an industrial control environment. Such a setup includes real-time controller, tools for real-time control design, programming, validation and optimisation, Supervisory Control and Data Acquisition (SCADA) system, and user friendly Graphical User Interface (GUI), while its application is considered for small standalone mobile devices, compact technologies, such as air-conditioning machines, up to large processes. Moreover, apart from sufficient performance, an appropriate control system platform must also contain standardised protocols and communication technologies allowing for a trouble-free implementation into buildings and other industrial control systems. Even though the system for BMS or BAS offered, for example, by companies that are mentioned in Section 1, seems to be sophisticated and sufficient to meet all of these requirements, there are several supplementary functions that shall be implemented in order to obtain a truly purpose-built and professional control system, featuring low-cost hardware, modern open-source, and free IT platforms and the possibility to be integrated into IoT or industrial IoT (IIoT) topology at the same time. In this section, we introduce the structure of a novel control system platform that is based on the DCU.

### 2.1. Structure of the Control System Platform

The following description of the DCU control platform is shown to meet the above requirements. This platform, developed by Prosystemy company [28] and GMCS in particular, is built on three pillars that provide a satisfying performance of the whole system. These can be defined, as follows:

- control algorithm design and implementation—including means to design control diagrams in a convenient software environment familiar to the developer;
- data processing and advanced supervision—together with database and advanced supervision procedures of the ASS package; and,
- global monitoring and visualisation—the visualisation of processes and design of GUI for a specific system operation and global management of ongoing processes.

**Figure 1:**
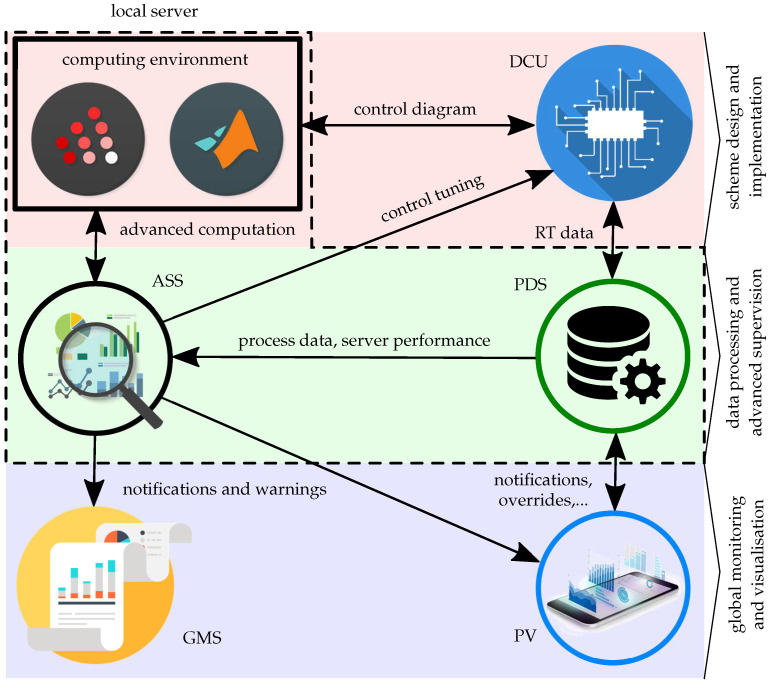
Simplified scheme of a global monitoring and control system. DCU—Dynamic Control Unit; ASS—Advanced Supervision Server; PDS—Process Data Server; GMS—Global Monitoring System; and, PV—Process Visualisation.

Figure 1 schematically represents a GMCS, where the three pillars are highlighted by colours. Note that the specific extension of the system lies in the integration of ASS and Global Monitoring System (GMS) into the GMCS structure, as indicated in Figure 1.

The DCU platform offers numerous advantages in comparison with other similar devices. For the design and development of control diagrams and algorithms, the well-known scientific computational platform MATLAB/Simulink or its open-source alternative Scilab/Xcos can be used. Ready-to-use libraries and toolboxes are available for the programmer and, thus, provide a comfortable software environment. The designed and possibly simulated control diagram can be compiled and uploaded to the DCU control unit (described in more detail in Section 2.2), which, together with the computing environment, creates the first pillar depicted in red.

The DCU control unit collects the real-time data from the process and transports them via a standardised communication technology into the Process Data Server (PDS), where the data are processed and stored in a database. PDS and the newly developed part, ASS, fall into the second pillar that is shown in green. In ASS, the operator can define all of the required procedures of supervision, which will be performed during operation of the system. This can involve, for example, signal analysis, frequency analysis, peak and outliers detection, threshold alerts, fault detection, or process model identification.

Because of more complex tasks, such as the computation of the secondary controller’s output in a cascade control scheme, closed-loop identification, but also fault detection and other procedures, ASS is in cooperation with the computing environment, where the result is evaluated and sent back to ASS for the purpose of implementation. For instance, controller tuning, i.e., the design of more convenient new parameters of a PID controller, is a good example. Based on the interaction between ASS and PDS, ASS is able to determine the status of further operation; whether it is a better or worse case in comparison with the previously recorded results. The most common example of result exploitation is notification of a user in the case of anomalies indicated by results.

The last layer of the control system, as illustrated by blue colour, represents the means of process visualisation, which provides a comfortable interface for process monitoring and it also ensures interaction between the user and system via control elements. This GUI helps the user to better understand the controlled process and provides him with all of the relevant notifications, alarms, override of values, and historical data available. Such a concept can be also used for monitoring of more complex processes in even more detail, creating the GMS, which can be very convenient for the operator during debugging or fault detection in a complex or even in several independent buildings or processes. Thus, GMS can provide operators and administrators with all of the necessary information via one interface.

In addition, the dashed line shown in Figure 1 represents the local server. This involves the development environment with programming and designing software for control algorithms and diagrams. The ASS package and PDS with real-time data that are stored in database is also included. The local server is, in this case, represented by a computing platform with Macintosh, Windows, or Linux operating system, i.e., a desktop PC, mini PC, industrial PC, or a laptop in hobby projects.

Therefore, the resulting scheme of the GMCS can be considered as a complete hardware/software setup for building control or other industrial systems, integrating the procedures of ASS, real-time control design, optimisation, controller tuning, SCADA, and global monitoring system.

### 2.2. Hardware and Software of Dynamic Control Unit

The entire GMCS is built on the Dynamical Control Unit, which can be generally used for development of various industrial control system as well as hobby applications, as outlined above. The DCU features the following ten specifications, which are also required for each fully-featured advanced control system:

(a) User-friendly graphical control design and programming environment.
(b) Process data recording into database system.
(c) Modern and universal HMI.
(d) Hard real-time process control (true sampling time starting at 1 ms).
(e) Provide all standard control elements in industrial and building management.
(f) Support advanced control algorithms and advanced control design techniques.
(g) Support controller networking.
(h) Support standard license-free communication protocol.
(i) Cost-effective hardware components.
(j) Open-source and platform independent software tools.

Most of the specifications that are listed above are commonly required in practice, but it is rare in a standard process control system to only use open-source, widely supported and platform-independent software tools. This is caused by the tendency of companies to develop and offer their proprietary solutions, which are often licensed to keep their know-how. However, the use of such open-source software combination, which supports at least Windows, Linux, or Mac operating system, helps, to a large extent, to avoid tailored designing and programming as well as to reduce expenses. Moreover, this feature increases modularity and flexibility of the control platform. Overall, the DCU control system platform fulfils all of the expected requirements on an advanced control system, while more information can be found on website of the Prosystemy company [28].

The control system platform consists of four main parts. The first is real-time control unit for execution of control tasks, the second is a computer, which provides logging of process data, HMI, and control design. The third part represents standard Local Area Network (LAN) as well as Wide Area Network (WAN), which are used in order to create a communication channel between the real-time control unit and the computer. The last part is the standard industrial network (Modbus RTU) used for connecting other control units or devices from other vendors.

In comparison with the classical Programmable Logic Controllers (PLCs), which are usually hardware structured into a centralised control topology and that involve one computational unit and a set of I/O modules, the DCU is a compact controller with a high-performance computational unit and certain number of physical I/Os. Therefore, each DCU can control a part of the process, such that it creates a distributed control topology. More details can be found online [28].

Two types of DCU controllers are available. Both of them also have an integrated RJ45 connector in order to provide UDP/TCP/IP service for processing data and real-time communication tasks, RS485 serial communication connectors for Modbus RTU service and power connectors. There is also an integrated pair of LED diodes to indicate voltage source and ongoing communication.

**Figure 2:**
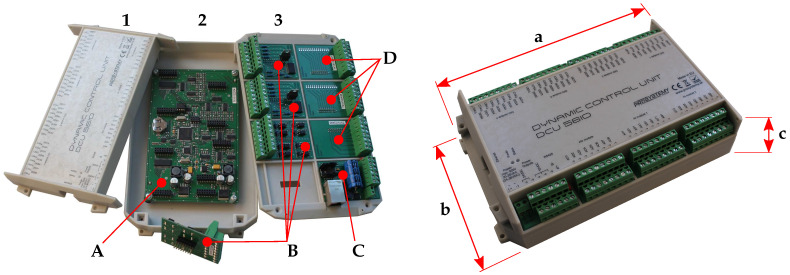
Dynamic control unit—DCU56IO (decomposed on the left). **A**—main DCU board; **B**—digital modules; **C**—power and communication module; **D**—analog modules; **1**—top cover; **2**—chassis; **3**—mid cover; **a**—width (230.2 mm); **b**—heigh (138 mm); and, **c**—depth (53.5 mm).

These two DCU controllers differ from each other in their size and number of I/O peripheries. The first, smaller one, is designated as DCU28IO and the second one as DCU56IO. DCU28IO is more embedded-application oriented, due to its size reduction from DCU56IO, and it offers fixed I/O peripheries layout only. In this unit, eight analog inputs, four analog outputs, eight digital inputs, and eight digital outputs are available. On the contrary, DCU56IO offers not only more I/O peripheries, but also their modularity. The DCU56IO consists of three plastic parts—chassis (**3**), mid cover (**2**), and top cover (**1**), as shown in Figure 2. These plastic parts are used to hold all of the electrical components. The main DCU board (**A**), along with the ARM-core microcontroller, EEPROM, and battery holder, and plenty of others relevant electrical components, also integrates multi-pin connectors for I/O modules. Up to 8 I/O modules can be used; one of them is power and communication module (**C**) and the other seven can be configured according to the designer’s and application’s requirements. One analog output module, two analog input modules (**D**), and four digital I/O modules (**B**) can be selected.

**Figure 3:**
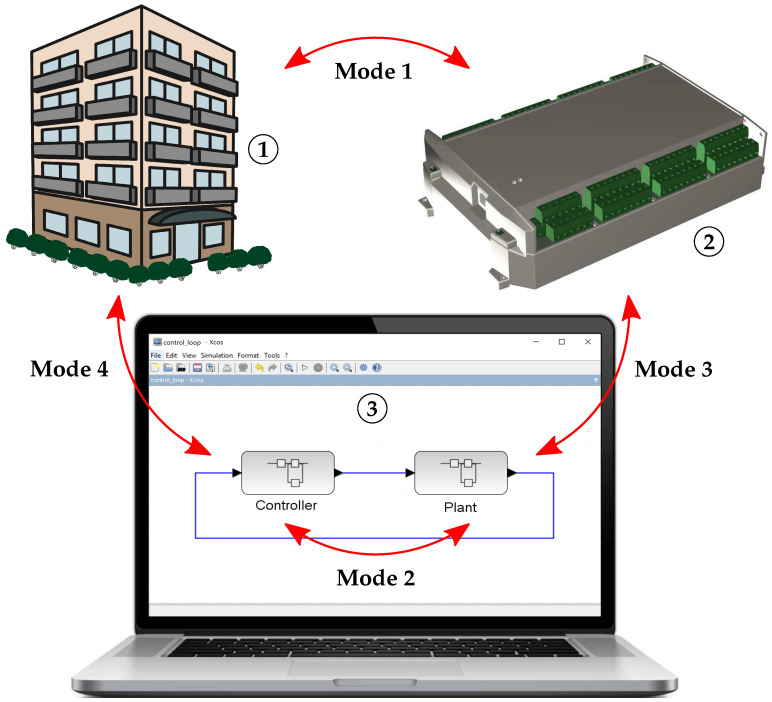
DCU control modes. 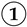
—real system/process; 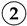
—DCU controller; and, 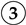
—host PC with simulation platform. NOTE: Red arrows connect parts, which cooperate in specific control mode.

DCU will be used for development as well as practical implementation and testing of the proposed ASS procedure described in the next section. Apart from this, it also supports controller implementation in several operation modes. Figure 3 illustrates four control modes of DCU that can be used in the control application design process as well as in a validation procedure. The actual DCU mode determines the behaviour of control application with respect to the physical I/Os. These control modes offer various possibilities to the designer, and they can be classified, as follows:

- **MODE 1**—real controller + real system—classic control mode, in which the real-time control application is executed inside the DCU controller and I/Os signals from real system are measured by physical I/Os channels of DCU;
- **MODE 2**—controller model + system model—classic simulation mode, where the controller and plant is designed in dynamic simulator environment, thus the control application execution in DCU is stopped and physical I/Os remain in the last state;
- **MODE 3**—real controller + system model—validation of the control algorithm (after mode 2), when the process model outputs are sent into DCU, where they are processed and evaluated, while output values are sent back to the process model in the host PC; and,
- **MODE 4**—controller model + real system—the real process is controlled, but the control application is executed inside host PC and DCU receives computed values of outputs and sends measured physical inputs back into the host PC.

The main advantage of mode 4 is that any block or function built or programmed inside the simulation platform (i.e., not only functions that are preprogrammed for DCU) may be used.

## 3. Advanced Supervision Server and Its Functions

The real-time control system, as described in Section 2.2, covers the first pillar in the concept of GMCS—scheme design, its validation, and implementation. To sum up, the real-time control scheme is designed and programmed in dynamic simulators, and then directly uploaded into DCU via UCC service. Consequently, the real-time data are collected into PDS (second pillar), from where they can be monitored and visualised in PV (third pillar) by means of an intuitive SCADA system.

The other two remaining parts of GMCS—ASS and GMS—make the system that is described above more intelligent and complex in terms of control optimisation, process monitoring and cost savings. These two parts form the main objective of our research interest. GMS monitors the health status of multiple controlled systems globally and offers all information on one GUI, being more comfortable and effective way of monitoring from the user, operator, or administrator point of view, as it has been already pointed out in Section 2. On the contrary, the ASS only analyses the selected processes locally and submits reports to the higher layer of the GMCS concept—SCADA and GMS.

The ASS with its tailored functions shall be used to solve these partial tasks in order to equip the control system with an universal tool for online analysis of a process, its closed-loop identification, and controller tuning. Moreover, the interfacing with a numeric computing environment, such as MATLAB, can be used to perform more complex and computationally demanding operations. The ASS functions can be divided into two groups:

- DCU I/O analysis—time-domain and frequency-domain analysis to detect anomalies, unwanted oscillations and switching frequencies, wear of system’s components, etc.; and,
- DCU process control analysis—closed-loop identification, controller tuning, and implementation of advanced control algorithms.

Note that the execution of these functions is performed in the background, so it is sufficient for the administrator or operator to first define a few required parameters and then he/she can monitor the process of evaluating a particular procedure or just wait for a warning message in PV or GMS.

### 3.1. Signal Analysis Procedure

One of the above functions has been designed, programmed, simulated, and tested in practice so far, as stated in Section 1. The underlying procedure belongs to the first group of ASS functions and is used for anomaly detection, including fault and illogical data trend detection in a specific process. For this purpose, the approach that is based on FFT is also used as a process of detecting signal peaks in the time-domain. The programmed advanced supervision analysis function, called signalCheck(), also searches for a sudden change over the operator defined threshold. This function operates in three main steps. The first is to read data from the input text or data file, which is automatically created by the UCC agent via a visualisation page, the second is to execute all of the commands for signal processing and analysis, and the last is to write the results to the output text or data file that is created and filled by signalCheck() function itself. Definition of required input parameters and boundary conditions represent a path to the location for output results file saving, critical values of the signal (period, amplitude and duration), sudden change interval and its critical amplitude. These critical parameters represent values that arise from the expected process behaviour, so the operator only needs to have basic technical knowledge in the signal analysis field. In addition to these settings, a matrix from time and signal value series data is contained in the input file, which is automatically filled by the UCC agent. Listing 1 shows the structure of the input data file for clarity.

**Listing 1:** Structure of the input data file.

**Row 1**—output folder path and file name, where to store results.
**Row 2**—informative message or any optional string.
**Row 3**—number of input parameters (matrices) for analysis.
**Row 4**—number of results (if previous results from analysis need to be considered).
*
**Row 5**—definition of matrix dimensions (number of rows and columns).
**Row 6**—the first row of matrix.
**Row 7…(x − 1)**—other rows of matrix (each row is on new line).
†
**Row x**—number of initial parameters.
**Row (x + 1)…(x + y)**—matrix dimensions and rows values—analogous to part between * and †.

Hence, the number of rows in the input file depends on the number of parameters, while each parameter is defined, as shown between * and † signs in Listing 1. After writing these parameters, the initial parameters are defined in the same way. The initial values are used in the case when the message of previous results contains ‘Initialisation’, and that is when the function is evaluated for the first time. An example of input data file can be found in Appendix A.

This data file is loaded and processed by the signalCheck() function programmed in Scilab 5.5.2 environment. The evaluation of the procedure begins by processing all information from the input data file and continues with determination of signal duration and its sampling time. Optionally, if the signal has an inverted logic in terms of time sequence, then it is reversed and rewritten. In the case of incomplete data matrix, an omission of measurement or the value is not expressed in the form of a number (NaN), the situation is solved by finding NaN samples and replacing these samples by the value that is computed as a mean of samples before and following them. The solution for two and more such samples located next to each other is also implemented. If this situation occurs, then the signal is broken in this place and two or more segments of the signal for further analysis are created. The same approach is used in order to solve the problem of incomplete data from time-continuity point of view. Based on this fact, the function segments the whole signal into time-continuous parts without any NaN values, which are sequentially analysed later. First, the lengths of each signal segments have to be checked and compared with a constant value determining the minimal size of data for analysis. In addition, the function skips segments of data, which have an outdated signal time. In other words, if the data sets are older than a user-defined time back from current time.

After the procedure of signal preparation for analysis and meeting all of the imposed requirements is done, the analysis itself may proceed. Within this stage, the fftAnalysis(), peaksAnalysis(), and changeAnalysis() functions, which are embedded in the main signalCheck() function and described next, are performed in a loop for every segment of the signal.

### 3.2. Main Functions of the Signal Analysis Procedure

The frequency analysis is executed by the fftAnalysis() function, which performs FFT algorithm for a particular moving time window with specified width, i.e., the windowing. Afterwards, the number of windows is specified for each signal segment that is based on its length, while half of the previous window width is added to all time windows in order to prevent the loss of information at the boundary between two consecutive time windows, as per the STFT algorithm. The number of windows is computed by dividing the segment length by a user-defined value of critical duration and the result is rounded down. The maximum magnitude with the corresponding frequency is determined in the loop for each time window and a criterion is computed. This criterion only depends on magnitude if the maximal determined magnitude is over critical frequency. Otherwise, the criterion is computed based on both magnitude and frequency. The highest value of the criterion is then expressed in percentage format in order to determine to what extent the user-defined critical parameters were attained.

Alternatively to this approach in frequency-domain, the solution in the time-domain has been programmed. To this end, the peaksAnalysis() function is used, in which the peaks over a defined threshold value (value of possible noise) are found by an algorithm that determines the differences in data vector with adequate sign (+/−) denoting the change in positive and negative direction, respectively. A peak and its magnitude can be detected by multiplying these changes in the data vector. Moreover, this depends on the user-defined value of threshold. Afterwards, the distances between these peaks are determined. Based on the distance between neighbours, the parts of signal are considered to be ‘normal’ or ‘critical’. If the peaks are close to each other, then it is considered as a critical state and the signal is analysed there. The period, amplitude, and time duration are defined and compared with critical values being defined by the user. Subsequently, the percentage criterion is calculated and compared with the percentage criterion that is determined by frequency analysis. These two results can be weighted by user defined weights for each technique of analysis and the bigger value is taken as indicator.

The last function embedded in signalCheck() performs an analysis of sudden change in time-series data. The sudden change search is executed by the algorithm of cumulative summation, which is a sequential analysis. The size of partial sums and difference between them is monitored. Thus, the biggest change in the signal data is represented by the highest difference between partial sums, and this is considered to be a result of t his function. Again, the percentage format of reaching the user defined threshold is applied.

Section 3.3 demonstrates the outputs of these functions and their structure.

### 3.3. Outputs, Other Features and Offline Simulation of the Procedure

All of the results provided by the above functions are processed and written to the output data file. The status of the procedure evaluation in form of a message is available to the user in the GMS and/or PV, where it is automatically loaded from the generated output file. Along with it, the numerical values in percentage format are present, as well as a timestamp (Unix timestamp is a sequence of characters representing date and time in seconds since 1st of January in 1970 at UTC), which declares the time of the maximum attained criterion. The structure of the output data file is given in Listing 2 and an example of the output file can be found in Appendix A.

**Listing 2:** Structure of the output data file.

**Row 1**—return message for user (status of the procedure evaluation).
**Row 2**—reached percentage value of criterion from *fftAnalysis()* and *peaksAnalysis()* functions.
**Row 3**—timestamp when the highest criterion was measured.
**Row 4**—attained percentage value of criterion from *changeAnalysis()* function.
**Row 5**—timestamp when the biggest change in signal was measured.

Another interesting and useful feature of the signalCheck() function, for debugging and more detailed information regarding an ongoing procedure in Scilab environment, has been programmed. The developer or programmer can choose between four modes of the function evaluation, which do not affect output data file structure, but more information in Scilab console are displayed or a graphical representation of the results is shown. Based on these options, the following four modes can be classified: minimalistic mode (debugging and graphical modes are disabled), debugging mode (debugging mode is enabled and graphical mode is disabled), graphical mode (debugging mode is disabled and graphical modes is enabled), and complex mode (debugging and graphical modes are enabled).

**Figure 4:**
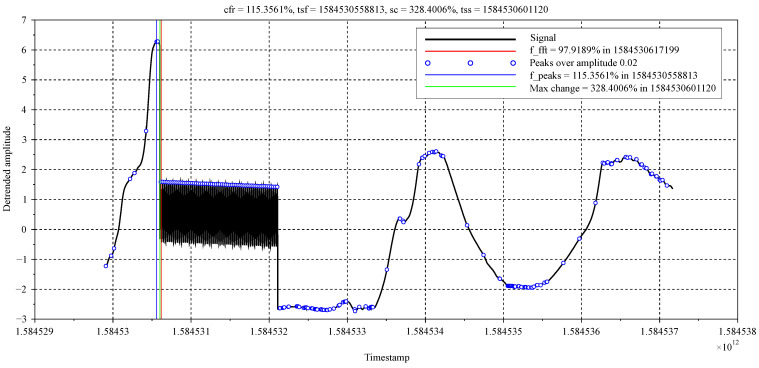
Graphical output from *signalCheck()* function simulation.

In case of the first, most simple mode, the function prints to the Scilab console only information regarding the completion of the procedure. In the second mode, a comprehensive statement of each relevant step in function is displayed in console. In case of graphical mode, a graphical window is generated, in which all important information are captured. In the case of complex mode, the graph, as well as detailed statement, are generated. An example of output statement generated during the procedure evaluation can be found in Appendix B and an example of graphical output from simulation of the function is shown in Figure 4 and more of them in Appendix C.

In this offline simulation, a dataset that was obtained by measuring air temperature in the HVAC system of a real building was used. The dataset was the result of approximately 120 min. long measurement and due to the creation of ‘critical state’, a harmonic signal was artificially imposed, so that the fault, which shall be detected, was created. This fault simulates, for example, sensor drop-out, problem with actuator, or similar. The harmonic signal was a sine wave with the parameters being set to the critical values given in input data file, so the output value should be 100%. The results that are represented by vertical coloured lines, as well as by numerical values in the legend and title, confirm this assumption, as shown in Figure 4. Thus, the beginning of the fault in the de-trended signal and the output values were determined.

## 4. Practical Case Study

After obtaining satisfactory simulation results and debugging the proposed procedure for signal analysis, i.e., signalCheck(), it was possible to verify its functionality and practically on a real system—an experimental two-storey family house situated in Bratislava, Slovakia, which the Prosystemy company had at its disposal for this purpose.

This residential building is a considerably complex system that includes several technologies. Apart from the central heating system and HVAC system, solar panels and indoor swimming pool automation are also involved. Because of the complexity of the system, six DCU units had to be integrated, while each DCU unit serves a certain subtask. This enables the access to a variety of signals from the sensors and actuators utilised in this system. Two of them, which represent the temperature trend, have been selected for signal analysis in our experimental verification. The first temperature signal comes from the central heating system and the second is from the HVAC system of this building.

### 4.1. Experimental Setup

The two temperature signals, selected for the experimental verification of the proposed signal analysis procedure, have been specified based on long-term experience of the building’s administrators, which implied that the chosen temperatures have a harmonic behaviour with a relatively high frequency. On the other hand, it is not so easy to reveal this kind of behaviour early within such a complex system. This would require lengthy observation of individual signals in the system, as well as analysis of historical data. Furthermore, the detection and early solution of problems caused by these anomalies is convenient not only for the user, but also for the process and technology in terms of less load on the components of the system. Therefore, the proposed procedures are particularly useful for the implementation of such a complex system.

**Figure 5:**
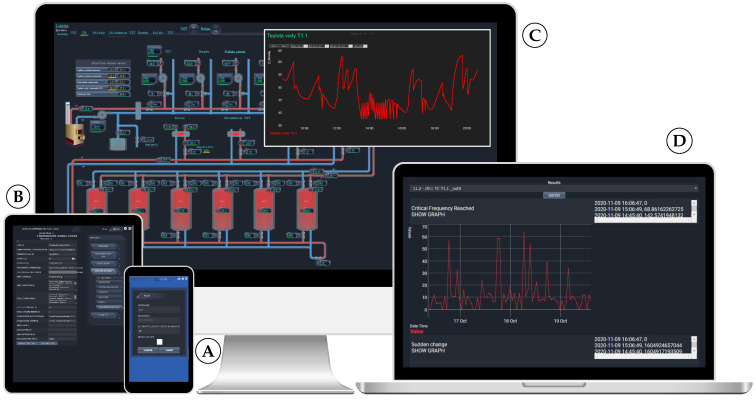
Visualisation accessible on any device with a web browser. 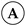
—mobile-phone; 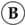
—tablet; 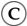
—desktop; and, 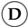
—laptop.

The signal analysis is initiated and its parameters are defined via a process visualisation interface, which can be accessed on any device with an installed web browser; examples are given in Figure 5. In order to make adjustments and set the ASS parameters, the administrator must first enter the login name and password (Figure 5A) in order to access the global monitoring and visualisation environment. Subsequently, the administrator may inspect the controlled system’s description, variable list, error and warning list, and visualisation of the entire system. For example, Figure 5C shows a visualisation of the central heating system of the family house subject to the experimental testing. Selecting any device (valves, pumps, etc.) further allows for changing their set-points. Moreover, a new window with signal data evaluation in time can be shown by clicking on the specific displayed value of the given signal (as shown in Figure 5C). The navigation menu also features the ‘Advanced supervision’ menu item, using which one may also perform the basic setup of the ASS and set parameters for the procedure (see Figure 5B). The specific settings of the procedure related our example can be found in Table 1. The most important values of parameters are typeset in bold font. The table lists the names of variables for analysis and critical values that shall not be exceeded. After entering the input parameter values in the visualisation environment, function aspcall.php and UCC agent take care of writing them into the input file for execution. Specifically, variable names that have been chosen to perform the analysis are *cfn:: TC T15.3_out0* and *cfn:: T1.1_out0*, denoting water temperature of outlet from the boiler and air temperature after the heating unit of the HVAC system, respectively.

The implementation procedure of the function considers up to 7200 s, i.e., a 2 h window of data for analysis, as shown in Table 1. The repeatability of the signal analysis is set to every 3600 s, i.e., every 1 h. Moreover, it is possible to set the priority of analysis execution by a value between 1 and 100, where a higher value implies higher priority. To sum up, in the presented experiment, the critical period was set to value 60 s, critical amplitude to 2 °C, and critical duration to 900 s. The sudden change threshold was set to value 3 °C and interval, within which the sudden change can occur, was chosen as 5 s. An output from this analysis can also be found in the given web interface under the label ‘Results’, as discussed in detail in the next section.

2

### 4.2. Experimental Results and Discussion

**Figure 6:**
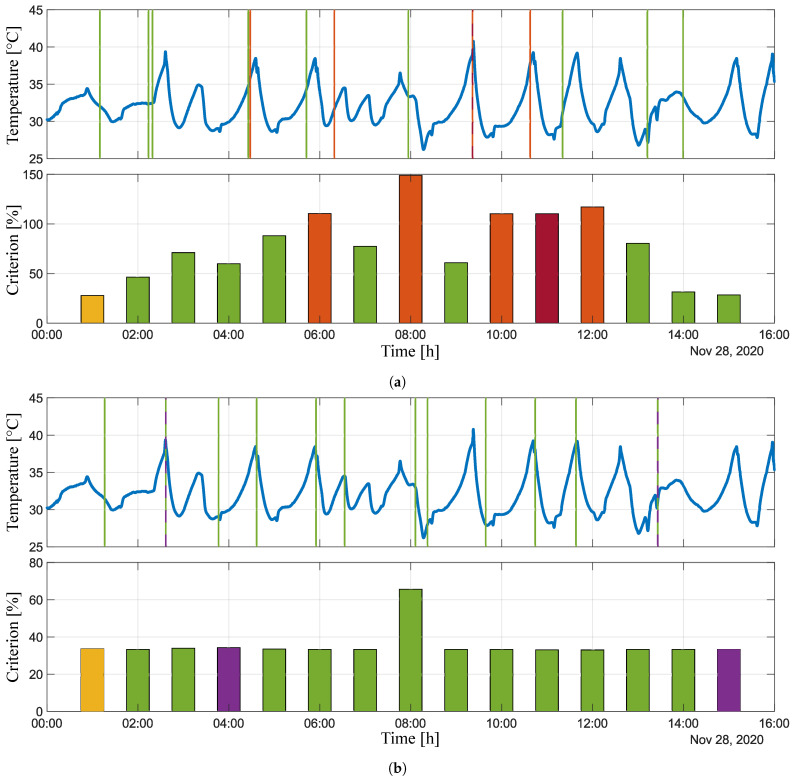
Experimental results of *cfn:: TC T15.3_out0* variable. (**a**) Signal evaluation and oscillation analysis. (**b**) Signal evolution and sudden change analysis.

**Figure 7:**
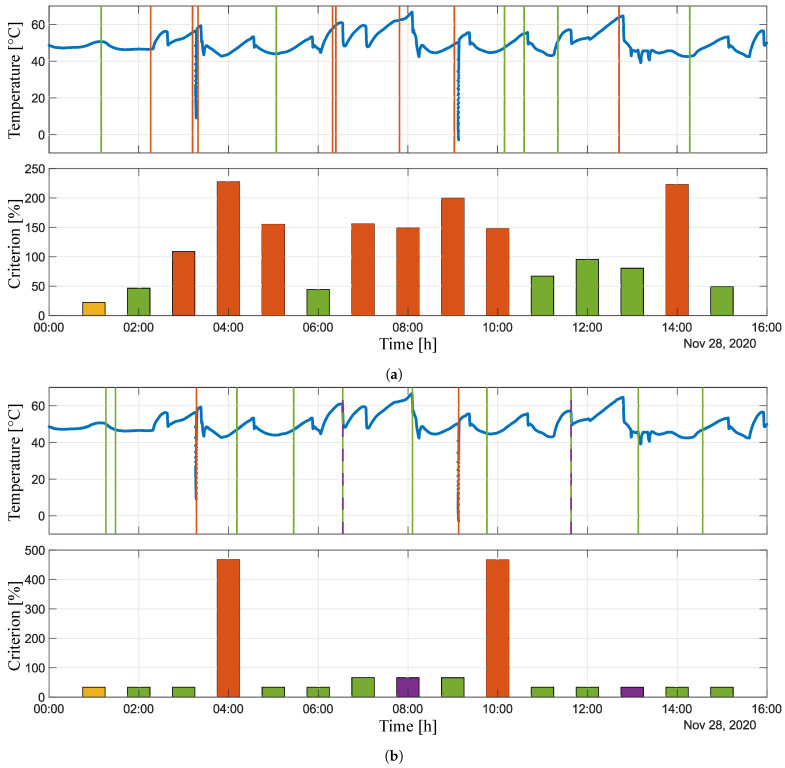
Experimental results of *cfn:: T1.1_out0* variable. (**a**) Signal evaluation and oscillation analysis. (**b**) Signal evolution and sudden change analysis.

The administrator can choose which signal analysis results for a given variable to display. Four graphs are plotted after selecting the variables. The first pair of graphs relates to the frequency analysis, where the first graph represents attained criteria value and the second graph provides information about the time at which the analysis detected the highest criteria values. The same is shown in the second pair of graphs, but here the sudden change analysis is displayed. An example of the experiment output is shown in Figure 5D. To make the results more clear, the MATLAB platform has been used for post-processing, thus the obtained data are presented in the MATLAB graphical window; see Figure 6 and Figure 7. Both of the signals, as well as the corresponding graphical and numerical outputs, are captured. Table 2 and Table 3 lists the corresponding numerical results. Note that the overall experiment lasted for sixteen hours on 18 November 2020.

Figure 6 shows the results from the analysis of the variable *cfn:: TC T15.3_out0*. This figure consists of two pairs of graphs. The first pair of graphs (Figure 6a) relates to the oscillation analysis, where the first graph illustrates signal evaluation in time and the bar graph below represents the maximum value of given criterion in [%] attained in specific times. The green colour highlights values below 100% and the red colour highlights those that exceeded this threshold. The vertical lines in the first graph capture the time when the highest value of the criterion was attained in a specific step of the analysis. The colours of these lines correspond to the colours in the bar chart. The yellow bar represents the result that is outside the displayed signal, and the dark red colour represents the result that is the same as the previous one, while the vertical line of this result is dashed. The second pair of graphs (Figure 6b) is the same, but it represents an analysis of sudden changes. The purple bars, analogously to the first pair of graphs, represent the same result as that obtained in the previous analysis step. Table 2 precisely lists quantified values of the attained criterion and the times at which the highest value of the criterion was detected in a given step.

The same holds for Figure 7 and Table 3, which present the results of the analysis of the second variable—*cfn:: T1.1_out0*. Based on the combination of graphical and numerical results, it can be stated that we obtained satisfactory results, because the portions of signal that approached the pre-defined critical values were reliably detected. Whether it is, for example, detecting the harmonic behaviour of a signal at the time from 6:19:14, when the criterion was attained to approximately 66% (see Figure 6a and Table 2), or the detection of a sudden decrease and increase of temperature in the second case (Figure 7b), where the criterion value was exceeded twice up to the value of approximately 467% (see Table 3).

Based on the performed practical experiments and obtained results, we can state that the assumptions from the simulations have been confirmed and the proposed procedure of advanced supervision is also reliable for long-term online deployment, since it has correctly detected and quantified any undesirable phenomena, not only within, but also after the presented experiment.

Nevertheless, certain limits and inadequacies have been observed. It is given that the Fourier transform cannot determine the duration of a given frequency in a signal, so the peaksAnalysis() function has been implemented and executed in parallel with the fftAnalysis() function. Within this experiment, the weights were set for the values of their results. The result of fftAnalysis() had a weight equal to 1, while the weight of the result of peaksAnalysis() was 0.75. As a part of further research, we intend to focus on possibly the last modification of this procedure, namely to adjust the signalCheck() function, so that the FFT is performed first and the analysis in the time domain follows directly in series, in order to determine the exact value of signal duration with a specific frequency.

## 5. Conclusions

In this paper, we introduced an approach to advanced process supervision and demonstrated the implementation of the proposed signal analysis procedure in practice—while using real smart building as a case study. The presented solution helps to ensure a continuous online monitoring of process behaviour and, thus, allows for the user to avoid a potential problem in time. This eventually represents a step towards low-cost operation of the building. Specifically, we proposed the procedure of signal analysis as a part of advanced supervision system. The ASS was designed as function package and one of its functions has been programmed and integrated into a novel, low-cost and open-source control platform DCU. The procedure serves for the analysis of any signal measured by the DCU unit, such that anomalies and illogical data trend can be detected. Functionality of the designed function was tested in offline simulations and then experimentally, while using a two-storey family house building. Based on the obtained results, we may conclude that the proposed system is fully functional and performed satisfactorily. Within further work, our aim is to extend the ASS package with identification algorithms as well as advanced control methods. Finally, the practical aim is to deploy the proposed solution in other buildings and industrial systems.

## Figures and Tables

**Table 1:** Experimental setup for *signalCheck()* procedure.

Parameter	Value
Label	Temperature Signal Check
Computational platform URL	http://127.0.0.1/ASS/ass/aspcall.php
Procedure call	signalCheck
Priority	50
Scheduled	EVERY 3600 SEC
For control variables	**cfn:: TC T15.3_out0**, **cfn:: T1.1_out0**
For control functions	
Input string	Temp.analysis jj
Input structure	Analyzed Temperature Signal: CONTROL_VARIABLE[**7200**SEC],
	Frequency Critical Period: **60**,
	Frequency Critical Amplitude: **2**,
	Frequency Critical Duration: **900**,
	Sudden Change Threshold [degC]: **3**,
	Sudden Change Interval [s]: **5**
Result structure	Critical Frequency Reached: REAL: 0,
	Sudden Change:REAL: 0,
	Time of Critical Frequency Event: TIMESTAMP: 0,
	Time of Sudden Change Event: TIMESTAMP: 0
Result record size	10
Result record resend	1
Visualisation notification	Crit. Frequency Reached: Oscillation is close to critical period.
Visualisation alarm	Crit. Frequency Reached: Oscillating is above critical period and amplitude.
GMS alarm	
GMS warning	
GMS notification	
Max execution time	30SEC

**Table 2:** Numerical results of *cfn:: TC T15.3_out0* variable.

Analysis Time	Oscillation Analysis		Sudden Change Analysis	
28/11/2020	Attained Criterion [%]	Attained in Time	Attained Criterion [%]	Attained in Time
01:59:51	46.3334	01:09:50	33.2977	01:16:06
02:59:51	71.0155	02:14:00	33.9134	02:36:49
03:59:51	59.8925	02:19:15	34.2351	02:36:49
04:59:51	88.0896	04:25:37	33.4914	03:46:29
05:59:50	110.4940	04:28:13	33.2420	04:36:42
06:59:50	77.3565	05:42:25	33.2640	05:55:09
07:59:50	148.9891	06:19:14	65.6254	06:32:50
08:59:51	60.8071	07:56:55	33.3015	08:06:15
09:59:51	110.2355	09:21:39	33.2728	08:22:16
10:59:50	110.2355	09:21:39	33.0811	09:39:07
11:59:57	117.1298	10:37:50	33.0036	10:44:25
12:59:57	80.3785	11:20:33	33.2893	11:38:14
13:59:57	31.4047	13:12:32	33.2516	13:26:10
15:00:10	28.4239	13:59:22	33.2770	13:26:10

**Table 3:** Numerical results of *cfn:: T1.1_out0* variable.

Analysis Time	Oscillation Analysis		Sudden Change Analysis	
28/11/2020	Attained Criterion [%]	Attained in time	Attained Criterion [%]	Attained in Time
01:59:49	46.3335	01:09:50	33.2977	01:16:06
02:59:49	108.8712	02:15:54	33.3104	01:28:54
03:59:49	227.4807	03:11:55	467.3365	03:17:12
04:59:49	155.0944	03:19:18	33.1927	04:11:20
05:59:48	44.0485	05:03:54	33.2763	05:27:12
06:59:48	155.5126	06:23:36	65.8576	06:32:54
07:59:48	148.9891	06:19:14	65.6254	06:32:50
08:59:49	199.7701	07:48:42	65.6596	08:06:07
09:59:48	147.9392	09:02:10	466.3525	09:07:55
10:59:48	66.8067	10:09:14	33.2872	09:45:42
11:59:55	95.6282	10:35:29	33.2592	11:38:14
12:59:55	80.3785	11:20:33	33.2893	11:38:14
13:59:55	222.7023	12:42:22	33.1799	13:08:13
15:00:08	49.0933	14:16:57	33.1973	14:34:18

## Data Availability

The data presented in this study are available on request from the corresponding author. The data are not publicly available due to privacy.

## Appendix A. Input and Output Data File Example

Input:
   C:/Users/minarcik/Desktop/sch/signalCheck_output_31.dat
Some string here if needed
6
1
7200,2
1584537167675,32.360452
1584537166670,32.360394
1584537165664,32.360343
1584537164659,32.357122
1584537163656,32.354222
…
1584529900994,29.928913
1584529899988,29.930158
1584529898983,29.931279
1584529897978,29.932288
1584529896973,29.933196
1,1
25.0
1,1
1.0
1,1
1500.0
1,1
2.0
1,1
20.0
Initialization
4
1,1
0
1,1
0
1,1
0
1,1
0

Output:
  MESSAGE: OK: Peaks signal analysis has been conducted. OK: Sudden change signal analysis has been conducted;
MATRIX_0: [75.95734313336];
MATRIX_1: [1584530612174];
MATRIX_2: [335.6069380092];
MATRIX_3: [1584532109604];

## Appendix B. Scilab Console List during the *signalCheck()* Procedure

  –>signalCheck(’signalCheck_input_13.dat’)
OK: Data have been read from file.
OK: Input data have been read.
OK: Previous results have been read.
OK: The signal is not continuous-time and has been divided into 5 segments
OK: A duration of the 1. segment is 7200.548 [s]
OK: The signal meets all the requirements and will be analysed…
OK: Signal analysis executed and the result is [cfr, tsf, sc, tss]:
!149.3251305613 1584530465256 328.4002204453 1584530601120 !
OK: Execution time of the signal analysis was 6.811 [s] (0.018-fftAnalysis + 0.029-peaksAnalysis + 6.764- changeAnalysis)
OK: A duration of the 2. segment is 0 [s] !ERROR: Signal length is shorter than 100 samples, so the analysis for this signal will not be executed! !
OK: A duration of the 3. segment is 0 [s]
!ERROR: Signal length is shorter than 100 samples, so the analysis for this signal will not be executed! !
OK: A duration of the 4. segment is 0 [s]
!ERROR: Signal length is shorter than 100 samples, so the analysis for this signal will not be executed! !
OK: A duration of the 5. segment is 33.913 [s]
!ERROR: Signal length is shorter than 100 samples, so the analysis for this signal will not be executed! !
OK: The final result is [cfr, tsf, sc, tss]:
!149.3251305613 1584530465256 328.4002204453 1584530601120 !
OK: Results have been written to the text file
   ans =
OK: FFT analysis has been conducted. OK: Sudden change signal analysis has been conducted.
